# AI-based methodologies for exoskeleton-assisted rehabilitation of the lower limb: a review

**DOI:** 10.3389/frobt.2024.1341580

**Published:** 2024-02-09

**Authors:** Omar Coser, Christian Tamantini, Paolo Soda, Loredana Zollo

**Affiliations:** ^1^ Unit of Computer Systems and Bioinformatics, Università Campus Bio-Medico di Roma, Rome, Italy; ^2^ Unit of Advanced Robotics and Human-Centered Technologies, Università Campus Bio-Medico di Roma, Rome, Italy; ^3^ Department of Diagnostics and Intervention, Radiation Physics, Biomedical Engineering, Umeå University, Umeå, Sweden

**Keywords:** artificial intelligence reinforcement learning, support vector machine, neural network, decision tree, lower extremeties

## Abstract

Over the past few years, there has been a noticeable surge in efforts to design novel tools and approaches that incorporate Artificial Intelligence (AI) into rehabilitation of persons with lower-limb impairments, using robotic exoskeletons. The potential benefits include the ability to implement personalized rehabilitation therapies by leveraging AI for robot control and data analysis, facilitating personalized feedback and guidance. Despite this, there is a current lack of literature review specifically focusing on AI applications in lower-limb rehabilitative robotics. To address this gap, our work aims at performing a review of 37 peer-reviewed papers. This review categorizes selected papers based on robotic application scenarios or AI methodologies. Additionally, it uniquely contributes by providing a detailed summary of input features, AI model performance, enrolled populations, exoskeletal systems used in the validation process, and specific tasks for each paper. The innovative aspect lies in offering a clear understanding of the suitability of different algorithms for specific tasks, intending to guide future developments and support informed decision-making in the realm of lower-limb exoskeleton and AI applications.

## 1 Introduction

Lower-limb rehabilitation is a field of great clinical relevance, dealing with the rehabilitation of individuals with motor disabilities in the lower-limbs a result of trauma, or neurological or musculoskeletal heath conditions. Shi et al. (2019); Zhou et al. (2021). Globally, in 2019, 2.41 billion individuals had conditions that would benefit from rehabilitation, contributing to 310 million YLDs (years of life lived with disability). This number increased by 63% from 1990 to 2019. The disease area that contributed most to prevalence was musculoskeletal disorders (1.71 billion people) (Cieza et al., 2020).

Traditionally, lower-limb rehabilitation has been conducted by human therapists through physical therapy including specific exercises. However, the integration of wearable robotic technologies, i.e., the lower-limb exoskeletons, and Artificial Intelligence (AI) is paving the way for the design of new tools and approaches to improve the quality of therapies and increase patients’ independence and mobility (Holzinger et al., 2019; Di Tommaso et al., 2023; Reddy, 2022).

Among all the devices purposely designed to assist patients’ lower-limbs, exoskeletons are wearable robots that closely interact with humans. Typically, they are mechatronic structures that operate alongside human limbs and increase human locomotory economy, augment joint strength, and increase endurance and strength (Tamantini et al., 2023a). Nowadays, exoskeleton devices are studied and employed in many application scenarios such as industry (Masood et al., 2016), space (Lovasz et al., 2017) and healthcare (Bortole et al., 2015; Pecoraro et al., 2022). Moreover, they can also be used in industry, for tasks such as heavy lifting or repetitive motion, reducing the risk of injury to workers (Herr, 2009; Pons, 2010). Exoskeletons can be used for rehabilitation, allowing patients with spinal cord injuries, stroke, or other conditions to regain mobility and improve their physical function. Currently, there is a large interest in the design of lower-limb-compliant exoskeletons aimed at gait rehabilitation or assistance (Zhang et al., 2016; Sanchez-Villamañan et al., 2019).

Lower-limb exoskeletons typically comprise a frame, actuators, sensors, and control systems that enable them to mimic human locomotion (Bhardwaj et al., 2021). Batteries or other energy sources can power them, and they can be controlled by a person’s actions, by a software-based controller, or by a combination of both (Talatian et al., 2021). Lower-limb exoskeletons are composed of actuators disposed on multiple joints, in particular the hips, ankles, or knees of the users. Each joint can assist different movements produced by an anatomical joint (Zhang et al., 2017). Therefore, the exoskeletons can be classified as follows according to the joint or joints that can be assisted during the robot-assisted gait.

• Hip Exoskeletons assist the hip joint connecting upper and lower-limbs, enabling a person to perform flexion/extensions, abduction/adduction, and medial/lateral rotation. These motions are necessary for a person to walk or run. Those exoskeletons have actuators placed on the users’ hips to enable a reduction of stress on hip and ankle muscles (Zhang et al., 2018).
• Most knee exoskeletons present only one DoF, needed for the motion on the knee flexion/extensions actions. In those cases, a soft inflatable cushion is typically used as an actuator. The inflatable part is placed behind the user’s knee for the reduction of weight of the exoskeleton. It uses a pneumatic system to inflate and deflate the component. The exoskeleton is inflated during the swing phase of the walking gait and deflated the gait cycle during walking (Celebi et al., 2013).
• Ankle exoskeletons have been developed to assist one degree of freedom. The ankle joint has four bones and three planar motions (three DoF). Plantar or dorsiflexion movement is a primary movement during the gait cycle that can be assisted through robotic devices (Gordon and Ferris, 2007).
• Multiple joints exoskeletons integrate more than one actuator to assist users during robot-assisted gait. They actuate a combination of joints implementing sophisticated control strategies, to face the increase in hardware complexity, and to produce a harmonious gait (Qiu-zhi et al., 2016; Franks et al., 2021; Kalita et al., 2021).

Within the realm of exoskeleton development, AI emerges as a fundamental cornerstone, actively contributing to various functional tasks in exoskeleton-assisted lower-limb robot-aided rehabilitation. These tasks include Robot Control (RC), Locomotion Classification (LC), Intention Detection (ID), and Human Joints Trajectory Prediction (HJTP). AI dynamically adapts to the wearer’s movements, seamlessly integrating the exoskeleton with the user. This adaptability enhances responsiveness and underscores the personalized nature of the interaction.

The synergy between AI and exoskeletons refines movement coordination, augmenting the potential for more efficient and tailored therapeutic interventions. AI methodologies optimize adaptability to tasks and users in RC. In LC, it improves adaptability to the environment and generalizes to unknown and unstructured settings (Tu et al., 2021). AI approaches contribute to advancing user action perception, enhancing system responsiveness, and promoting the customization of responses, leveraging data from wearable sensors and cameras (Wu et al., 2018; Tamantini et al., 2023b), in ID and HJTP applications, respectively. This review aims at elucidating how the application of AI, as indicated by these acronyms, adds value to various aspects of exoskeleton-assisted lower-limb robot-aided rehabilitation, supported by evidence in scientific literature. Through literature analysis, we intend to provide a comprehensive understanding of the specific contributions of AI in different contexts of exoskeleton-assisted lower-limb robot-assisted rehabilitation.

The rest of the paper is structured as follows: Section 2 presents the reviews already published to better highlight which is the novelty of this specific contribution to the scientific literature. Section 3 presents the methodology implemented to carry out the review of the scientific literature. Section 4 details the results of the review. In particular, the AI-based solutions exploited in exoskeleton-assisted gait are analyzed in depth. Section 5 discusses the major outcomes of the literature analysis providing advantages, limitations, and future vision of this research topic. Lastly, Section 6 summarizes the paper’s contribution and Appendix 1 lists all the acronyms used in the text.

## 2 Current review

In recent years, the field of robot-aided rehabilitation has attracted considerable attention, resulting in a proliferation of scholarly works. The existing body of literature encompasses several comprehensive reviews delving into the application of exoskeletons in lower-limb rehabilitation (Prakash et al., 2018; Khera and Kumar, 2020; Mu et al., 2021; Harris et al., 2022). However, this review distinguishes itself by uniquely focusing on the integration of advanced Artificial Intelligence (AI) methodologies within this domain, presenting a novel and innovative contribution.

Khera and Kumar (2020) meticulously explore the role of machine learning in gait analysis, identifying Support Vector Machine (SVM) as a prominent classifier based on their analysis of 43 distinct studies. Similarly, Mu et al. (2021) delves into the application of AI in rehabilitation, specifically concentrating on objective assessment methods. Their investigation encompasses various AI-driven parameters, such as trajectory error features, joint angles, joint angular velocities, and surface Electromyography (sEMG) signal features, laying a foundation for our emphasis on advanced AI techniques. Prakash et al. (2018)’s extensive survey comprehensively covers gait analysis across diverse domains, presenting various machine-learning approaches alongside associated datasets. While informative, this survey sets the stage for our unique contribution, which integrates cutting-edge AI methodologies. Lastly, Harris et al. (2022)’s recent survey explores the application of AI to human gait, identifying six key areas of focus. While valuable, their work underscores the evolving landscape, leaving room for novel insights, particularly in the convergence of AI methodologies with lower-limb rehabilitation.

In essence, our review’s distinctive emphasis on the integration of advanced AI methods within the context of exoskeleton-assisted lower-limb robot-aided rehabilitation positions it as a unique contribution to the existing literature. By championing this innovative approach, our review offers fresh perspectives and insights that hold the potential to steer future advancements in this critical field.

## 3 Methods

A comprehensive literature search, updated until October 2023, was conducted across Google Scholar, Scopus, and Web of Science databases. The search utilized keywords and their combinations, including (Lower-Limb*) AND (Robot* OR *Exoskeleton*) AND (*Artificial Intelligence* OR *Machine Learning* OR *Deep Learning*). Remarkably, our preliminary literature analysis underscored a notable recurrence of specific algorithms, revealing that Reinforcement Learning (RL), Support Vector Machine (SVM), Neural Network (NN), and Decision Tree (DT) stood as the exclusive focal points. This initial scrutiny served as the foundation for our subsequent investigation, providing a clear direction for the inclusion of these predominant algorithms in our review. Consequently, more specific searches were performed using combinations like (Lower-Limb*) AND (Robot* OR *Exoskeleton*) AND (*Reinforcement Learning* OR Neural Network* OR Support Vector Machine* OR Decision Tree*) to refine the focus of the research.

**FIGURE 1:**
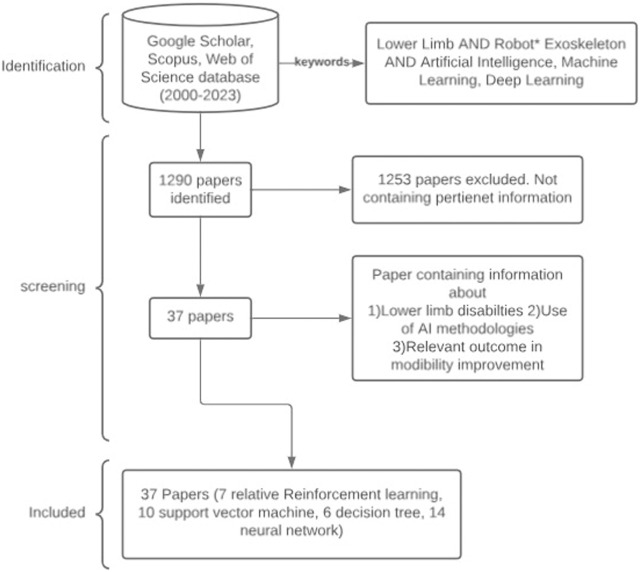
Flowchart of the search and inclusion process.

Figure 1 presents the PRISMA flowchart constructed to highlight the review process. The inclusion criteria for this review are summarized as follows

• The study should be published between 2000 and 2023.
• The study should be published in reputable journals or international conferences employing a peer-review process. This stringent selection ensures the validity and reliability of the works considered for inclusion in the analysis.
• The study should report a novel algorithm tested in simulation environments, as well as involve participants ranging from healthy subjects to those with lower-limb motor disabilities, such as spinal cord injuries and/or stroke.
• The study should focus on the application of AI-based methodologies for exoskeleton-assisted lower-limb rehabilitation, explicitly declaring the presented algorithm elucidating the specific conditions necessary for replicating the training process, and implementing the proposed approach. The input features, the hyperparameters, and the working conditions of the presented approach should be clearly stated to consider the study eligible.

Among the resulting literature works, 40 papers have been selected blue after removing duplicates and applying the inclusion criteria. The studies obtained from the literature analysis can be described by analyzing the AI algorithm implemented in each study.

## 4 AI approaches for exoskeleton-assisted rehabilitation of the lower limb

**FIGURE 2:**
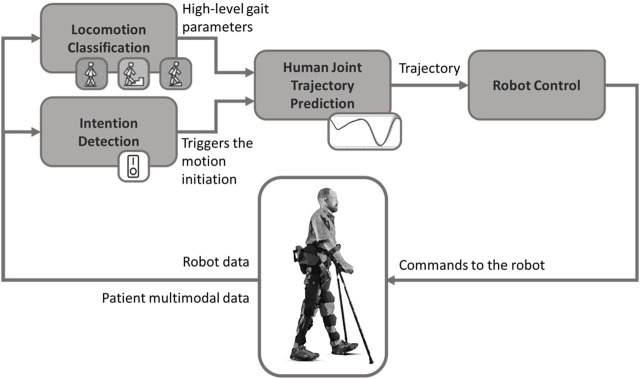
Scheme of the main functional blocks of a lower-limb exoskeleton for rehabilitation that can exploit AI methodologies.

Figure 2 reports the schematic representation of the main functional blocks of a lower-limb exoskeleton for rehabilitation that can exploit AI methodologies. In this schematic overview, the central focus is on the exoskeleton user, around whom a comprehensive set of data is gathered from both the robotic system and multimodal patient monitoring. This influx of information is then directed into two pivotal modules: LC and ID. The former aims at discerning high-level parameters related to walking, such as step-phase and terrain characteristics, while the latter focuses on identifying the user’s intention to initiate walking. The outputs from these modules converge into the trajectory generation system, a critical component tasked with predicting the joint angles necessary for the exoskeleton to generate a walking pattern customized to the individual’s anthropometric dimensions. Ultimately, the culmination of this predictive information guides the RC module, which takes charge of the actual actuation system. By leveraging the current configuration of the robot and aligning it with the desired movement, this control module plays a pivotal role in executing and optimizing the exoskeleton’s response to ensure a seamlessly tailored and adaptive walking experience for the user. The modules depicted in this block diagram can be implemented using traditional approaches or innovative methodologies based on AI, which is the central focus of this review.

Tables 1–3 presents a comprehensive overview of the research papers reviewed in this paper. Each row includes paper references, objectives, features, models, performance, subjects (H: healthy, P: pathological), robots, and different application scenarios. In particular, we include four main scenarios, which are RC, LC, ID, and HJTP. By structuring the information in this manner, the table offers a comprehensive overview of the included papers and their content. The table is ordered with respect to methodologies and year, and it is divided into four sections as this section does. Indeed, the 37 papers reviewed here are grouped into four categories according to the AI methodology adopted, namely, Reinforcement Learning (RL), Neural Networks (NN), Support Vector Machine (SVM), and Decision Tree (DT). In Table 1, “non-robot” means that the proposed AI algorithm was fully designed and validated in a simulation environment without any validation into a real exoskeletal platform.

**TABLE 1:** AI-based solutions implemented for exoskeleton-assisted lower-limb robot-aided rehabilitation.

Paper	Objective	Feature	Model	Performance	Subjects	Robot	Applications
*Reinforcement Learning*							
Bingjing et al. (2019)	Human-robot interactive control	Joint angle and human forces	SARSA	Err:0.2 rad	1H	CARR robot	RC
Khan et al. (2019)	Compliance control of a robotic walk assist device	Joint position angle and velocity	Qlearning	≤ Err:0.08 rad	Sim	Sim	RC
Xu et al. (2020)	RL for robotic mirror therapy	Motion Trajectory	Q-network	Err: 1.05 rad	5P (paretic leg)	Research prototype	RC
Luo et al. (2021)	RC of lower exoskeleton for squat assistance	Joint position, velocity and centre of pressure	Proximal policy optimization	Err:0.05 rad	Sim	Sim	RC
Peng et al. (2021)	RC for lower-limb rehabilitation exoskeleton	Joint angle	actor-critic neural network	Err:0.1 rad	Sim	Sim	RC
Rose et al. (2022)	deep-RL for exoskeleton gait control	Force and position	deep q-learning	Err:0.01 rad	Sim	Sim	RC
Luo et al. (2023)	deep-RL control of lower-limb rehabilitation exoskeleton	Joint angle, velocity	deep q-learning	Err:0.1 rad	1H, 3P (Different neuromuscular disabilities)	Research prototype	RC
*Neural Networks*							
Lim et al. (2010)	Gait parameter prediction	Cadence, stride length, walking speed	NN: MLP	deviation of 0.03%	50H	Non-robot	LC
Luu et al. (2011)	Waveforms planning	Anthropometric data	NN: MLP	0.98 a	15H	Non-robot	HJTP

**TABLE 2:** Continue of AI-based solutions implemented for exoskeleton-assisted lower-limb robot-aided rehabilitation.

Paper	Objective	Feature	Model	Performance	Subjects	Robot	Applications
Jung et al. (2015)	Gait phase classification	Human forces	NN: MLP	0.98 a	7H	ROBIN-H1 Exoskelton	LC
Liu et al. (2016)	Knee joints modelling	Hip, knee angle	NN:Long-short term memory	≤ Err:0.30 rad	20H	SIAT-Exoskelton	HJTP
Błażkiewicz and Wit (2018)	Joint angle simulation	Ankle, knee and hip angle	NN: MLP	0.94 a	34H	Non-robot	HJTP
Yang et al. (2019)	NN controller for Lower-limb Rehabilitation Exos	Emg	NN: Radial basis function	Err: ≤ 1⋅ 10^–3^ rad	Sim	Sim	RC
Xiong et al. (2019)	Joint moment prediction	Emg and joint angles	NN:Elastic	0.96 a	8H	Non-robot	HJTP
Yingxu et al. (2019)	Control of Lower-Limb Rehabilitation exos	Hip knee and joint torque and speed	NN: MLP	Err:0.90 rad	Sim	Research prototype	RC
Fereydooni et al. (2020)	Control of lower-limb exoskeleton	sEMG	NN: wavelet	0.01 m	4H	Research-prototype	RC
Shi et al. (2020)	Trajectory tracking	Hip knee and ankle angle	NN: MLP	Err: ≤:0.02 rad	Sim	Research-prototype	RC
Zhou et al. (2020)	Individual gait generation	Hip,knee, ankle position and force	NN: recurrent NN	Err: ≤ 0.16 rad	137H	Non-Robot	HJTP
Tang et al. (2021)	Evaluation of postoperative rehabilitation of patients	Angular velocity	NN: recurrent NN	1 a	36H	Non-robot	LC
Wang et al. (2021b)	Data augmentation and trajectory prediction	Hip and knee angle	NN: GAN	0.88 a	5H	Non-robot	RC
Lin and Sie (2023)	NN controller for lower-limb robotic	Hip and knee angle	NN: MLP	Err:0.03 rad	10H	Research-prototype	RC
*Support Vector Machine*							
Ceseracciu et al. (2010)	SVM classification of locomotion using emg	Emg	SVM	0.95 a	3H	Non-robot	LC
Shen et al. (2013)	Motion intent recognition for wearable lower device	Hip and Knee position	SVM	0.96 a	1H	Research prototype	ID

**TABLE 3:** Continue of AI-based solutions implemented for exoskeleton-assisted lower-limb robot-aided rehabilitation.

Paper	Objective	Feature	Model	Performance	Subjects	Robot	Applications
Li et al. (2017)	Gait recognition of Lower-Limb rehabilitation robot	Horizontal distances	SVM	0.95	3H	Non-robot	LC
Lin et al. (2017)	Lower-Limb rehabilitation	Emg	SVM	0.99 a	8H	Research prototype	RC
Ziegler et al. (2018)	Classification of gait phases using emg	Emg	SVM	0.96 a	8H	Non-robot	LC
Wang et al. (2019)	Human gait recognition	feet pressure, waist and calf movement	SVM	0.965 a	5H	Non-robot	LC
Ma et al. (2019)	Gait phase classification	Hip and knee angle	SVM	0.86 a	10H	Research prototype	LC
Guo et al. (2020)	Control strategy for lower-limb rehabilitation	sEmg	SVM	0.95 a	10H	SIAT Exoskeleton	ID
Ge et al. (2022)	Lower-limb movement recognition	sEmg	SVM	0.907 a	6H	Non-robot	LC
*Decision Tree*							
Guo and Jiang (2015)	Walking gait identification	Force sensors, knee angle, angular velocity	Decision tree	0.99 a	1H	Research-prototype	LC
Ren et al. (2018)	Patient-specific gait training	Anthropometric data	Decision Tree	Err:0.08 rad	113H	Research-prototype	LC
Thongsook et al. (2019)	Gait Phase Recognition	Hip knee position, walk velocity	Decision tree and NN and NARX	1, 0.987 and 0.948 a	1H	Research-prototype	LC
Imura et al. (2021)	Stroke patient identification	Age, sex and stroke type	Decision tree	0.85 a	481P (stroke)	Non-robot	LC
He (2022)	Control of motion rehabilitation robot	Hip, knee and ankle position	Decision Tree	0.997 a	10H	Research-prototype	RC
Zhang et al. (2022)	Gait Deviation Correction method	CGA curves	Decision Tree	Err: ≤ 0.11 rad	15H	Research-prototype	RC

### 4.1 Reinforcement learning

Reinforcement learning (RL) is a category of AI used for training an intelligent agent to perform tasks or achieve goals in a specific environment by maximizing the expected cumulative reward. It has three main components: the action space, the state space, and the reward, indeed the agent moves around the state space by taking an action, and the one that maximizes the reward is the favorite (Kaelbling et al., 1996).

In Bingjing et al. (2019), the authors propose a method for controlling a lower limb exoskeleton. The goal is to assist patients in a way that is safe, effective, and tailored to the individual patient’s needs. The exoskeleton assists both lower-limbs, each with two rotational degrees of freedom. The exoskeletal robot is driven by a pneumatic proportional servo system, which allows for accurate motion control. To enable effective human-robot interaction, the authors adopt an adaptive admittance model. Admittance control is a method used to regulate the interaction between a robot and the environment, i.e., the patient. The admittance model used in this study is adaptive so that it can adjust its parameters based on the patient’s needs and performance. The authors design an adaptive law for the admittance parameters using a sigmoid function and an RL algorithm. The agent is the exoskeleton, and the environment is the patient. By using RL to optimize the admittance parameters, the authors obtained individualized parameters that are suitable for each patient’s specific needs. Joint angle error and human-robot contact force are selected to form the state space, the action is selected among four possibilities that correspond to up, down, left, and right movements. The *ϵ*-greedy policies are applied to the on-policy method, meaning that with a probability of 0.5 the maximal estimated action value is chosen. The experiment was conducted to verify the feasibility of the interactive control system with the hip and knee joint angle data in the Clinical Gait Analysis (CGA) database as reference trajectories and is conducted on the prototype of a gait rehabilitation training exoskeleton. The database is established by capturing a large number of motion information of normal people while walking by the 3-D Motion Capture System of Northern Digital Technologies Inc. The system has been tested on 1 healthy subject with an error of 0.2 rad (radiant) (Bingjing et al., 2019).

The proposed scheme in Khan et al. (2019) is an optimal adaptive compliance control for a robotic walk assist device. The approach is based on bio-inspired RL and is completely dynamic-model-free. The scheme uses joint position and velocity feedback, as well as sensed joint torque applied by the user during walking, for compliance control. In particular, the RL system has a state vector containing hip and knee joint angular position and velocities and the action is selected via an actor-critic system. The effectiveness of the controller is tested through simulations using an exoskeleton walk-assisting device model, returning angular error ≤ 0.08 rad for both the tested DoFs. In particular, is used RWAD developed in the SimMechanics toolbox (Matlab/Simulink).

Xu et al. (2020) proposed a master-slave exoskeletal system for mirror therapy to transfer therapeutic training from the patient’s functional limb to the impaired limb using a wearable robot in Xu et al. (2020). The IL mimics the action prescribed by the FL with the assistance of the exoskeleton, stimulating and strengthening the injured muscles through repetitive exercise. The RL approach was used in the human-robot interaction control to enhance rehabilitation efficacy and guarantee safety. The study incorporated multi-channel sensed information, including the motion trajectory, muscle activation (expressed via skin surface electromyography signals), and the user’s emotion (shown as facial expressions), into the learning algorithm. The RL approach was realized by the normalized advantage functions algorithm. Furthermore, the study developed an exoskeleton with magnetorheological actuators, and clinical experiments were conducted using the proposed system to verify the performance of the framework. In particular, five hemiplegic patients were enrolled in the experiments. During the experiment, the patient exerts force on the intact limb to drive the master robot, and the impaired limb tries to actuate its own muscles to complete the gait task. In particular the classical Q-learning, in continuous action spaces with deep neural networks is used. Overall, the study demonstrated the potential of a master-slave robotic system with RL to enhance the effectiveness and safety of mirror therapy in lower extremity rehabilitation. The incorporation of multi-channel sensed information and the use of MR actuators in the exoskeleton design can also provide valuable insights for developing more advanced rehabilitation systems (Xu et al., 2020).

The study Luo et al. (2021) proposed a new motion controller for a lower extremity rehabilitation exoskeleton using RL. The exoskeleton is designed for collaborative squatting exercises and equipped with ankle actuation on both sagittal and front planes and multiple foot force sensors to estimate the center of pressure, a critical indicator of system balance. The proposed controller takes advantage of CoP information by incorporating it into the state input of the control policy network and adding it to the reward during learning to maintain a well-balanced system state during motions. To improve the robustness of the controller, the study also used dynamics randomization and adversary force perturbations, including large human interaction forces during training. The effectiveness of the learning controller was evaluated through numerical experiments with different settings to understand if the learning process can generate feasible control policies to control the exoskeleton to perform well-balanced squatting motions if the learned control policies are robust enough under large random external perturbation and to sustain stable motions when subjected to uncertain human-exoskeleton interaction forces from a disabled human operator. The study demonstrated the potential of RL-based motion controllers for lower extremity rehabilitation exoskeletons, particularly in terms of efficiency, stability, and robustness. The proposed controller’s ability to incorporate CoP information and handle large human interaction forces during training can be valuable in real-world rehabilitation settings, where maintaining balance and stability is critical (Luo et al., 2021).

The paper Peng et al. (2021) proposed an innovative approach for adaptive control of lower-limb exoskeletons. The proposed approach combines policy iteration, RL, and event-triggering mechanisms to achieve online learning and adaptation while reducing the number of control updates. In particular, an adaptive online learning structure, namely, Actor-Critic Neural Network, is exploited in the Event-triggered Optimal Control framework, The proposed EtOC is then verified on numerical simulation, and tested of a real lower-limb rehabilitation exoskeleton with a final error of 0.1 rad. Overall, the proposed approach presents a promising direction for adaptive control of lower-limb rehabilitation exoskeleton robots (Peng et al., 2021).

The paper Rose et al. (2022) proposed a new event-triggered control strategy based on RL and policy iteration for a lower-limb rehabilitation exoskeleton. The proposed approach integrates an event-triggering mechanism and a novel event-triggered tuning law with an actor-critic neural network for online learning and adaptation. The experimental results show that the proposed method reduces the number of control updates while maintaining a guaranteed control performance compared to traditional time-triggered control methods. The experiment has been simulated with an error of 0.01 rad These methods can potentially enhance the adaptability and efficiency of control systems while reducing the computational burden and energy consumption (Rose et al., 2022).

In Luo et al. (2023), the authors proposed a deep RL-based robust controller for a lower-limb rehabilitation exoskeleton. The controller is trained using a decoupled offline human-exoskeleton simulation training with three independent networks. The goal is to provide reliable walking assistance against various and uncertain human-exoskeleton interaction forces. The controller acts on a stream of the LLRE’s proprioceptive signals, including joint kinematic states, and predicts real-time position control targets for the actuated joints. To handle uncertain human interaction forces, the control policy is trained intentionally with an integrated human musculoskeletal model and realistic human-exoskeleton interaction forces. Additionally, domain randomization is employed during training to increase the robustness of the control policy to different human conditions, with different neuromuscular disorders. The trained controller provides reliable walking assistance to patients with different degrees of neuromuscular disorders without any control parameter tuning. The system has been tested on 3 pathological subjects and 1 healthy with a final error of 0.1 rad (Luo et al., 2023).

To wrap up we can say that Reinforcement learning has gained attention in the exoskeleton-assisted rehabilitation field thanks to its ability to learn optimal control strategies through the interaction with the environment by committing errors and receiving a reward based on its entity and to solve problems that with classical analytical methods would be hard to solve. We saw how deep reinforcement learning is still poorly investigated even if in other fields has been shown to surpass classical reinforcement learning. In particular deep reinforcement learning provides a new solution for rehabilitation robot trajectory planning and control strategies with a high accuracy (Wang et al., 2021a). Given the high quantity of algorithms available the choice strictly depends on the given problem (Arulkumaran et al., 2017).

### 4.2 Neural networks

Neural networks are a subset of machine learning and are at the heart of deep learning algorithms, they are comprised of node layers, containing an input layer, one or more hidden layers, and an output layer. Each node is connected to another and associated weight and threshold. If the output of any individual node is above the specified threshold value, that node is activated, sending data to the next layer of the network, otherwise, no data is passed along the next layer (LeCun et al., 2015).

This paper Lim et al. (2010) discusses the use of an MLP to predict natural gait parameters for an individual based on their age, gender, body height, and body weight. The MLP is trained to output a suitable walking speed and cadence for the given subject. The study evaluates the efficiency and accuracy of the MLP in predicting the desired outputs for two different setups. In the first setup, the MLP is trained specifically for slow walking speed conditions. In the second setup, the MLP is trained for both slow and normal walking speed conditions. The input features of the model are cadence, stride length, and walking speed. The model has been tested on 50 healthy subjects. The proposed approach was capable of returning accurate prediction with a deviation of 0.03%. The study demonstrates the potential of using MLP for predicting natural gait parameters for an individual, which can be useful in fields such as rehabilitation and sports science (Lim et al., 2010).

In Luu et al. (2011), the authors present a new model for generating joint angle waveforms of the lower-limb during walking using gait parameters and lower-limb anthropometric data as input. The locomotion data is captured using a motion capture system with passive markers, and the waveforms of lower-limb joint angles are calculated from the experimental data. The waveforms are then decomposed into Fourier coefficients, which allows for easier waveform analysis. They designed an MLP to predict the Fourier coefficient vectors for specific subjects and desired gait parameters. Assessment parameters such as correlation coefficient, mean absolute deviation, and threshold absolute deviation are calculated to examine the quality of the MLP prediction. The constructed waveforms from predicted Fourier coefficient vectors are compared with the actual waveforms calculated from experimental data using the assessment parameters mentioned above. The results show that the constructed waveforms closely match the experimental waveforms based on the assessment parameter outcomes, demonstrating the potential of using MLP to accurately predict joint angle waveforms of the lower-limbs during walking, in particular, the system has been tested on 15 healthy subjects with an accuracy of 0.98. This can be useful in fields such as biomechanics and rehabilitation where understanding and optimizing human movement is important.

The paper Jung et al. (2015) proposed a method for gait phase classification in lower-limb exoskeleton robots, using sensor signals from foot sensors with force sensing registers, as well as the orientation of each lower-limb segment and the angular velocities of the joints. The authors investigated MLP and NARX. During the experiment, the subject wore the ROBIN-H1 exoskeleton and walked on a treadmill following specific rules. The performance of the proposed classifiers was evaluated using offline and online evaluations based on four criteria: Classification Success Rate, Max Continuous Error Width, Mean and Standard Deviation, and Number of unstable regions. The results showed that the NARX-based method exhibited satisfactory performance in replacing foot sensors as a means of classifying gait phases. The feature used as input is human forces. The system has been tested on 7 healthy subjects with an accuracy of 0.98. The results suggest that this approach could lead to more accurate and reliable gait phase classification, which could be useful for improving the performance and safety of exoskeletons (Jung et al., 2015).

The paper Liu et al. (2016) proposes a method called Deep Rehabilitation Gait Learning (DRGL) for modeling the knee joints of lower-limb exoskeletons. The method leverages Long-Short Term Memory (LSTM) to learn the inherent spatial-temporal correlations of gait features. With DRGL, abnormal knee joint trajectories can be predicted and corrected based on the wearer’s other joints, without requiring complex kinematic and dynamic models for the human body and exoskeleton. The main advantage of DRGL is that the new recovery gait pattern is not only in accordance with the healthy walking gait but also includes the wearer’s own gait profile. To prove the effectiveness of DRGL, the authors obtained a new recovery gait from DRGL based on “pathological gait,” which was obtained by having a healthy subject imitate knee injury. The experiments demonstrated that the subject could walk normally with the SIAT lower-limb exoskeleton in the new recovery gait pattern generated by DRGL. The system has been tested on 20 healthy subjects. The proposed neural architecture is capable of reconstructing human trajectories with an angular error ≤ 0.30 rad and capturing the key gait features with high consistency. This suggests that DRGL is a promising method for improving gait rehabilitation using lower-limb exoskeletons. Overall, the paper presents a novel approach for gait rehabilitation using deep learning techniques. The proposed DRGL method could have important implications for the design and development of lower-limb exoskeletons that can help patients recover from knee injuries or other conditions affecting their gait (Liu et al., 2016).

The paper Błażkiewicz and Wit (2018) aimed at developing a neural network that accurately simulates the changes in the angle of the ankle, knee, and hip joints during the gait cycle, and to use it to simulate the impact of a restricted range of ankle and hip joint angle changes on the progression of the knee joint angle. The study involved 34 young healthy students, and gait kinematics data were collected using the Vicon system, which was then analyzed with an MLP. The results showed that the developed MLP was able to accurately simulate the progression of joint angles of lower-limb motion with an accuracy of 0.94, and its simulation of the impact of restricted ankle and hip joint angular ranges on the knee joint indicated that the braking phase is critical. The study highlights the potential of NNs as a useful research method in clinical biomechanics and suggests that further research in this vein could expand our understanding of compensatory functions in the lower-limbs. The findings could be useful in the design and development of lower-limb exoskeletons and other assistive devices, as well as in the rehabilitation of patients with lower-limb injuries or conditions affecting their gait (Błażkiewicz and Wit, 2018).

The paper Yang et al. (2019) presents two control schemes for exoskeletons to conduct trajectory tracking tasks in the presence of unmodeled dynamics of the exoskeleton, interaction between human and exoskeleton, and additional disturbances. The first controller presented is purely NN-based (Radial basis function NN), and adopts a combined error factor (CEF) to enhance human safety by improving transient response. The CEF consists of the weighted sum of tracking error and its derivative. The second controller is developed based on a combined scheme of repetitive learning control (RLC) and radial basis function NN, where the add-on RLC is used to learn periodic uncertainties that contribute to the repetitive motion of the exoskeleton leg. The stabilities of the controllers are proved rigorously in a Lyapunov way. The study highlights that while the pure NN controller can deal with periodic and non-periodic uncertainties simultaneously, the main feature of exoskeleton motion during rehabilitation therapy, namely, the repetitiveness, is fully ignored, and this could degrade the tracking performance. The proposed control method is shown to achieve a significant control effect with remarkable transient performance in comparison to the other methods used in the simulation. The input data used was EMG and the system has been simulated achieving an error ≤ ⋅10^–3^ rad. Overall, the study demonstrates the potential of NN-based control methods for improving the performance and safety of lower-limb exoskeletons in rehabilitation therapy. The results could be useful in the design and development of advanced control strategies for assistive devices in rehabilitation and other applications (Yang et al., 2019).

The paper Xiong et al. (2019) presents a novel method for predicting joint moments using a small number of input variables selected by Elastic Net and MLP. The method is tested on experimental data collected from healthy subjects running on a treadmill at different speeds. The results show that the method can accurately predict joint moments with only 5-6 EMG signals as input, with a normalized root-mean-square error (NRMSE) lower than 7.89% and a cross-correlation coefficient (p) between the predicted joint moment and multibody dynamics moment. This method can effectively reduce the input variables required for joint moment prediction, which may facilitate real-time gait analysis and exoskeleton robot control in motor rehabilitation (Xiong et al., 2019).

This paper Yingxu et al. (2019) describes a study on the application of a bionic control method based on a Central Pattern Generator (CPG) to control the lower-limb exoskeleton for rehabilitation purposes. The authors improved the Hopf oscillator using the Dynamic Hebbian Learning algorithm and built a CPG oscillator network to generate gait signals to control the lower-limb exoskeleton. The study aimed at improving the performance and adaptability of the exoskeleton by matching it with the control signals produced by the human body during motion cycles. It included wearing tests on patients, and the results obtained by the simulation experiments of gait curves of different motion modes showed that the CPG bionic control exoskeleton could effectively control the lower-limb exoskeleton and perform rehabilitation exercises. The system has been simulated, the input data was hip, knee, and joint torque plus speed the final angle error was 0.90 rad. The study provides valuable insights into the application of CPG-based control methods for exoskeleton rehabilitation and highlights the potential for improving the performance and adaptability of lower-limb exoskeletons for rehabilitation purposes (Yingxu et al., 2019).

The paper Fereydooni et al. (2020) proposes an intelligent control method for a lower-limb exoskeleton using sEMG and human force-based dual closed-loop control strategy. The method aims to adaptively control the exoskeleton to better function in the rehabilitation field. The proposed approach has several contributions. First, it uses a wavelet neural network (WNN) to obtain the desired trajectory of patients based on the sEMG signal. Second, it modifies the reference trajectory by the variable impedance controller (VIC) based on the sEMG and human force. Third, it uses a model reference adaptive controller (MRAC) with parameter updating laws based on the Lyapunov stability theory to force the exoskeleton to track the reference trajectory. The experiment results show that the proposed approach efficiently decreases the trajectory tracking error and adapts the reference trajectory to synchronize with the patients’ motion intention. The model reference controller can outstandingly force the exoskeleton to track the reference trajectory. The final system has been tested on 4 healthy subjects with an error ≤ 0.01 m. The proposed method is expandable to other applications in the rehabilitation field and has the potential for designing an intelligent control for rehabilitation purposes.

The paper Shi et al. (2020) dealt with the recovery of a patient’s limb and is driven to follow a planned trajectory during rehabilitation training. The accuracy of trajectory tracking is essential for effective rehabilitation training. PID control is a conventional method for trajectory tracking, but due to the dynamic model uncertainties and lack of good adjustment ability, it may not be sufficient. Therefore, the authors combined RBF neural network and PID control to improve the accuracy of trajectory tracking. The Magnetorheological (MR) damper and motor for actuation. The control is simulated in Simulink, and the trajectory tracking errors under PID control and RBF-PID control are compared. The results obtained through the simulation experiment show that RBF-PID control has better anti-interference performance, which improves the flexibility of movement, real-time, and stability of trajectory tracking during the therapy. The system has been simulated with a final error of 0.02 rad.

The paper Zhou et al. (2020) proposed an individualized gait pattern generation method based on a recurrent neural network (RNN) for creating a function mapping from body parameters and gait parameters to a gait pattern. The proposed method is trained on the largest gait data set of this kind, which consists of 4,425 gait patterns from 137 healthy subjects (Zhou et al., 2020). The RNN is proficient in series modeling and can generate gait patterns at continuously varying walking speeds and stride lengths. The proposed model’s experimental results indicate that it reduces the errors in ankle, knee, and hip measurements by 12.83%, 20.95%, and 28.25%, respectively, compared to the previous state-of-the-art methods, in particular with a generalized regression neural network (Luu et al., 2014) and a gaussian process regression (Yun et al., 2014). It has significant implications for personalized rehabilitation training, including designing customized rehabilitation programs, identifying patients at risk of falls, and assessing the effectiveness of the rehabilitation program. The paper’s findings demonstrate the potential of using RNNs for gait pattern generation in rehabilitation (Zhou et al., 2020).

The paper Tang et al. (2021) proposed an RNN for rehabilitation evaluation. This method extracts gait characteristic parameters of patients with different ages, disease types, and disease courses by learning existing clinical gait data. The algorithm uses repeated data iteration to simulate the corresponding gait parameters of patients on 36 healthy subjects. Experiments show that the trained ANN algorithm has a high accuracy rate when compared to human raters for most of the data (82.2%, Cohen’s I kappa = 0.743). The algorithm also has a strong correlation with improved Ashworth scores as assessed by human raters (r = 0.825).

The paper Wang et al. (2021b) proposes a two-stage attention model placed inside an LSTMs-based encoder-decoder architecture for predicting human gait trajectories using a combination of real and synthetic gait data. The authors use a Generative Adversarial Network (GAN) with temporal Convolutional Neural Network CNNs to generate synthetic human gait data (ratio 4:1 synthetic vs. real data that retains the dynamics of real gait data. They collected real human gait data from five healthy subjects using a NOKOV optical motion capture platform. The authors compare the performance of their GAN model with a traditional LSTM model and found that the attention mechanism (MLP) had a higher capacity for learning dependencies between historical gait data to accurately predict the current values of the hip joint angles and knee joint angles in the gait trajectory. The results obtained by the collection of five healthy subjects’ gait data indicate that GANs-based data augmentation can synthesize realistic-looking at multi-dimensional human gait data. The predicted gait trajectories based on the historical gait data can be used for gait trajectory tracking strategies. This paper demonstrates the potential of using synthetic data to augment real data and improve the performance of machine learning models for human motion analysis tasks. The final accuracy is about 0.88 (Wang et al., 2021b).

This study Lin and Sie (2023) focuses on the development of a lower-limb exoskeleton for rehabilitation purposes, using artificial neural networks to improve its control system. Firstly, the authors testes the proposed lower-limb robotic exoskeleton robot (LLRER) using a PID control with an iterative learning controller. However, they found that the knee part of the LLRER, which uses PAM actuation, does not perform very well due to nonlinearity. To compensate for this nonlinearity, the researchers used an MLP control based on the inverse model trained in advance. They also used particle swarm optimization (PSO) to optimize the PID parameters based on the MLP architecture. The results show that the MLP with PID control (PSO tuned) performs the best among the three controllers. The average Mean Absolute Error (MAE) of the left knee joint is 0.03 rad and the average MAE of the right knee joint is 0.024 rad, tested on 10 healthy subjects. During rehabilitation tests, the controller of MLP with PID control was found to be suitable, and its versatility for different walking gaits was verified during human tests. The researchers found that the establishment of the inverse model does not need to use complex mathematical formulas and parameters for modeling, making it more accessible. Additionally, the use of PSO to search for the optimal parameters of the PID and the architecture diagram and control signal given by the MLP compensation with the PID control effectively reduced the error.

In conclusion we saw how NN can address several tasks from RC, and LC to HJTP. Thanks to the ability to learn patterns from raw data they are particularly useful for real-life applications. The choice of the NN architecture and dimension strictly depends on the problems (Hunter et al., 2012) often is easier to test several architectures developed based on theoretical knowledge of the problem and choose the best performing one (Warrier and Amuru, 2020). Lastly, it is worth observing that a big dataset is needed to properly train the NN and obtain good accuracy.

### 4.3 Support vector machines

This section presents the application of SVM to exoskeleton-assisted lower-limb robot-aided rehabilitation. SVM is a supervised learning method used for both classification and regression. The name came from the data points called support vectors that are closer to the hyperplane and influence the position and orientation of the boundary in the feature space. Using these support vectors, it is possible to maximize the margin of the classifier (Gunn et al., 1998).

In Ceseracciu et al. (2010), the authors explore the use of an SVM for identifying locomotion intentions from surface electromyography (sEMG) data. The study uses a phase-dependent approach, which is based on foot contact and foot push-off events, to contextualize muscle activation signals. The study demonstrates good accuracy on experimental data from three healthy subjects. The classification accuracy is also tested for different subsets of EMG features and muscles, to identify the minimal setup required for the control of an EMG-based exoskeleton for rehabilitation purposes. The study shows that SVM can be used to accurately identify locomotion intentions from sEMG data. The phase-dependent approach used in this study helps to contextualize muscle activation signals, which improves the accuracy of the classification. The system has been validated on 3 healthy subjects with an accuracy of 0.95 The study also highlights the importance of selecting the optimal subset of EMG features and muscles for the control of EMG-based exoskeletons for rehabilitation purposes (Ceseracciu et al., 2010).

The paper Shen et al. (2013) proposed a motion intent recognition method to control a wearable lower extremity assistive device intended to aid stroke patients during activities of daily living or rehabilitation. The primary goal is to identify the user’s intended motion based on sensor readings from the limb attached to the assistive device to execute the right control actions to effectively aid the user in his intended action. To this end, the study collected a database of 1 healthy subject performing various motion tasks. The features of the signals are extracted, and Principal Component Analysis is performed to reduce the number of dimensions. Using the transformed signal, a multi-class SVM with a Radial Basis function kernel is trained to classify the different motion patterns. A Nelder-Mead optimization algorithm is used to select the appropriate parameters for the SVM. An offline classification result of a healthy subject performing a series of motion tasks while wearing the LEAD shows that the proposed method can effectively recognize different motion intents of the user. The test results show that the SVM can correctly classify each motion pattern with an average accuracy equal to 0.9580 %± 0.04%. The study demonstrates the potential of using motion intent recognition methods to control wearable assistive devices intended to aid stroke patients during ADL or rehabilitation. The proposed method can effectively recognize different motion intents of the user, and the high accuracy rate of the SVM classification suggests that this approach may have practical applications in real-world settings.

The paper Wu et al. (2016) proposed two machine learning models for predicting gait phases from spatial and spatiotemporal perspectives using joint angle data collected from four goniometers and plantar pressure distribution data from three force-sensitive resistors. The two models are SVM optimized by particle swarm optimization algorithm and nonlinear autoregressive models with external inputs. The results of the experiment show that both models are capable of predicting gait phases, but NARX outperforms SVM in terms of accuracy since it utilizes FSR data to correct the wrong predictions. The system has been validated on 10 healthy subjects with an angular accuracy of 0.087 rad The authors suggest that it is better to predict gait phases based on both space and time dimensions simultaneously. The paper presents an interesting approach to predicting gait phases using machine learning techniques and demonstrates the effectiveness of incorporating spatiotemporal features in the prediction process (Wu et al., 2016).

The paper Li et al. (2017) discusses the use of a Kinect sensor and a lower-limb exoskeleton to provide targeted rehabilitation training. The Kinect sensor is positioned in front of the robotic exoskeleton and is used to acquire horizontal distance data from markers placed on the patient’s body during the rehabilitation training. This data is used to identify different patients using an SVM. By identifying different patients, the system can provide personalized and targeted rehabilitation training to each patient based on their specific needs. This approach is more effective than a one-size-fits-all approach as it takes into account individual differences in gait and movement patterns. The system has been validated on 3 healthy subjects with an accuracy of 0.95. Overall, the paper demonstrates the potential of combining technology, such as the Kinect sensor along with exoskeletons to improve the effectiveness of rehabilitation training and provide personalized care to patients.

The paper Lin et al. (2017) proposes a real-time electromyography-triggered controller for a pneumatic artificial muscle-actuated lower-limb exoskeleton. The proposed controller is designed to make the rehabilitation task controllable by the patient’s movement intention. To this good, the EMG signals of the patient’s muscle are captured and identified using the discrete wavelet transformation technique to acquire the feature vectors of the EMG signals. The optimal multicomponents of features are chosen based on the experimental results, and SVMs are studied to improve the classification performance. To implement the closed-loop control system for the rehabilitation robot with the movement-intention trigger control, they also used the MyRIO controller. This system allows the patient’s movement intention to be accurately identified by EMG feature extraction, ensuring the safety and performance of the proposed system. The system has been tested on 8 healthy subjects with a final accuracy of 0.99. Overall, the paper demonstrates the potential of using EMG-triggered control for a lower-limb exoskeleton, which can help provide personalized care to patients by enabling the exoskeleton to respond to the patient’s movement intention in real time (Lin et al., 2017).

The paper Ziegler et al. (2018) proposes a method for classifying the stance phase and swing phase during healthy human gait based on the muscle activity in both legs using SVM. The paper introduces a novel EMG feature calculated from the bilateral EMG signals of muscle pairs, showing promising results with classification accuracies of up to 0.96% tested on 8 healthy subjects. In particular, all used motion data were taken from the HuMoD database which is an open-source human motion dynamic database (Wojtusch and von Stryk, 2015). The proposed method could potentially have practical applications in the design of rehabilitation devices or assistive technologies that are intended to aid individuals with gait impairments. The use of SVMs in this study suggests that this approach may be effective for other classification tasks in the field of rehabilitation, and the introduction of the novel EMG feature may inspire further research into the development of new features for classification tasks.

The paper Wang et al. (2019) describes the development of a multi-sensor fusion gait recognition system for accurately controlling exoskeleton movement. The system acquires plantar pressure and acceleration signals of human legs, and in the experiment, the pressure signals of both feet and movement data of the waist left thigh, left calf, right thigh, and right calf of five test subjects were collected. The test lasted 3 min and consisted of standing, going up the stairs, going down the stairs, going up and down the slope, and walking on level ground. The study investigated six different gaits, including standing, level walking, going up the stairs, going down the stairs, going down the slope, and going down the slope. The authors demonstrated that the SVM outperformed Multilayer perceptron (MLP), and radial basis function (RBF) neural networks models. The study also analyzed the different sliding window sizes of the SVM algorithm. The results showed that the SVM algorithm had the highest recognition rate with an average recognition accuracy equal to 0.965%, tested on a total of 5H subjects. The accurate recognition of human gait provides a good theoretical basis for the design of a control strategy for a lower-limb exoskeleton. The study’s findings could have potential applications in the development of assistive devices for people with mobility impairments and in the design of exoskeletons for industrial and military use.

The study Ma et al. (2019) aims to improve gait phase classification in an exoskeleton using only the angle of hip and knee joints by introducing a kernel recursive least square algorithm to build a classification model that considers the adaptation of unique gait features. Additionally, an assist torque predictor based on the KRLS algorithm is also developed. The study compares the performance of the KRLS model with two other commonly used gait recognition methods, MLP and SVM, using gait data collected from 10 healthy volunteers wearing the exoskeleton. The results show that the KRLS classification accuracy is on average 3% higher than MLP and SVM, with a testing average accuracy of 0.86%. The KRLS algorithm also performs twice as well as MLP in assisting torque prediction experiments. Furthermore, the KRLS algorithm is demonstrated to be stable, robust, and able to generalize well to different datasets. The study suggests that the KRLS algorithm is a promising method for improving gait phase classification and assisting torque prediction in exoskeletal robots (Ma et al., 2019).

In Guo et al. (2020) the use of SVM in this application is based on its ability to classify and recognize patterns in complex datasets, such as the sEMG signals collected from the surface of human muscles. The proposed method involves the collection of sEMG signals from the human body during different motion postures, such as walking, standing, or sitting. These signals are then processed and analyzed using an SVM classifier to recognize the intended motion posture of the wearer. The output of the SVM classifier is then used to plan the moving gait of the exoskeleton, and the decoding intention signal controls gait switching. To ensure the stability of the planned gait during movement, the researchers also analyzed the stability of the exoskeleton during the execution of different motion postures. The experimental results showed that the SVM-based method of decoding sEMG signals for human motion intention and controlling exoskeleton gait switching had good accuracy and real-time performance. The system has been tested on 10H subjects with an accuracy of 0.95 The application of SVM to lower-limb exoskeleton has significant potential for aiding in the rehabilitation of patients. By using sEMG signals to identify human motion intentions and control the exoskeleton’s movement, patients can complete rehabilitation training more safely and quickly. Additionally, the use of SVM in this application may have implications for other fields that require the real-time recognition and classification of complex datasets (Guo et al., 2020).

The paper Ge et al. (2022) describes an experiment for lower extremity action recognition using sEMG signals. The experiment was designed based on the principle of human lower extremity force generation, and the aim was to classify five common lower extremity actions Natural walking, sitting, standing up, and going up, and down the stairs.). In particular, the subject performed specific movements, and a set of raw sEMG data was collected. The authors extracted the sEMG time-domain feature used to train an SVM classifier. The results of the experiment showed that the SVM classifier achieved an average accuracy rate equal to 0.9066% evaluated from 6 healthy subjects, which verified the effectiveness of the experimental design. Overall, the paper presents a novel approach to lower extremity action recognition using sEMG signals and demonstrates the effectiveness of using time-domain features and an SVM classifier. The results of the experiments will have potential applications in fields such as rehabilitation, sports training, and human-machine interaction (Ge et al., 2022).

To conclude, even if SVM is an old technique it is still used for a high range of tasks like RC, LC, and ID. Thanks to its ability to perform well in high-dimensional space with an excellent accuracy it remains a valid competitor (Mayoraz and Alpaydin, 1999). The long training time makes it difficult to use on big datasets and for the non-linear separation the choice of the kernel is not always easy (Karamizadeh et al., 2014; Ma and Guo, 2014).

### 4.4 Decision tree

A DT is a non-parametric supervised learning algorithm, used both for classification and regression tasks. It is characterized by a hierarchical, tree structure, which consists of a root node, branches, internal nodes, and leaf nodes (Charbuty and Abdulazeez, 2021).

The paper Guo and Jiang (2015) presents a method for identifying the sub-phases of gait in human-machine coordinated motion using an exoskeleton. The authors introduce a sensor layout that includes shoe pressure sensors, knee encoders, and thigh and calf gyroscopes to measure the contact force of the foot, knee joint angle, and angular velocity. To differentiate between human lower-limb motion and human-machine coordinated motion, the authors divide the sub-phases of the gait cycle: double standing, right leg swing, and left leg stance, double stance with the right leg front and left leg back, right leg stance and left leg swing, and double stance with the left leg front and right leg back. The authors use a C4.5 decision tree algorithm to fuse the sensor information blue and through classification identify the sub-phases of the gait cycle. Based on simulation results, the proposed algorithm guarantees identification accuracy. The experimental results performed on 1 healthy subject verify the gait division and identification method. The input features are force knee angle and angular velocity and the final accuracy is about 0.99. Moreover, the authors suggest that the proposed method can make hydraulic cylinders retract ahead of time and improve the maximal walking velocity when the exoskeleton follows the person’s motion (Guo and Jiang, 2015).

The paper Ren et al. (2018) proposed a method based on anthropometric features for predicting patient-specific gait trajectories. The authors noted that human gait pattern are closely related to anthropometric features, but this relationship has not been well-researched. The proposed method uses the Fourier series to fit gait trajectories and represent gait patterns by the obtained Fourier coefficients. The authors use human age, gender, and 12 body parameters (Age, Height, Mass, Gender, Thigh length, Calf length, Bi-trochanteric width, Bi-iliac width, ASIS breath, Knee diameter, Foot length, Malleolus height, Malleolus width, Foot breadth) to design the gait prediction model. To make the method applicable to lower-limb exoskeletons, the anthropometric features are selected using an optimization method based on the minimal-redundancy-maximal-relevance criterion. The relationship between the selected features and human gaits is modeled using a random forest predicting patient-specific gait trajectories. The system has been validated on data collected in a previous study (Yun et al., 2014) from 113 healthy subjects (Luu et al., 2014) achieving an error of 0.08 rad.

The paper Thongsook et al. (2019) compares the performance of three algorithms for recognizing three different gait phases of a passive lower limp exoskeleton: C4.5 decision tree, MLP, and NARX. The gait phases are stance, swing, and push, and the algorithms use two IMU sensors on the hip and knee joint position and two FSR sensors in a self-made shoe to provide four inputs. Test data containing the pressure of shoes contacting the ground, knee angles, and knee angular velocities is collected at different walking speeds, and the experimental results show the classification success rate of each algorithm when trained with different pattern sizes. The system final accuracy is equal to 1 for C4.5, 0.98 for MLP, and 0.948 for NARX, tested on 1 healthy subject The experiments help to determine the most suitable algorithm for recognizing different gait phases in real-time applications (Thongsook et al., 2019).

The work Imura et al. (2021) aimed at identifying the factors that affect home discharge after stroke inpatient rehabilitation, including both functional and environmental factors, using the machine learning method. The authors note that while the importance of environmental factors for stroke patients to achieve home discharge has been discussed, there are limited studies on the application of the decision tree with various functional and environmental variables to identify stroke patients with a high possibility of home discharge. To address this gap, the authors collected a private dataset including information on 481 stroke patients’ functional status and environmental factors, such as living arrangements and social support, and used the three classification and regression tree (CART) models to identify the factors that predict home discharge with a final accuracy of 0.85. The results showed that functional factors, such as motor function and cognitive function, were the most important predictors, followed by environmental factors, such as living arrangements and social support (Imura et al., 2021).

The paper He (2022) proposes a motion control system for lower-limb exoskeleton for postoperative rehabilitation training, which is based on human posture information. The system has several functions, such as active/passive training mode control, movement posture and EMG signal acquisition, WiFi communication, and safety protection. The training process is recognized and analyzed using random forest and linear regression. The experimental results performed on 10 healthy subjects show that the random forest algorithm has better performance in motion recognition than the linear regression algorithm with an accuracy of 0.997. The features used in the system are hip knee and ankle positions. The developed control and monitoring system can be controlled by Android and can realize the intelligent analysis of the training process through the monitoring signals in the training process (He, 2022).

The gait deviation correction method proposed in Zhang et al. (2022) aims to decrease the deviation of the wearer’s gait when using a lower-limb exoskeleton. By using the clinical gait analysis curve as a reference trajectory and incorporating body feature parameters, the gait correction model modifies the input trajectory of the exoskeleton to reduce gait deviations. In particular, the gait deviation correction method is based on the XGboost algorithm a commonly used algorithm in the industry due to its highly efficient implementation of the gradient boosting algorithm. To train the algorithm CGA curves have been used. The results performed on 15 healthy subjects showed that the correction led to closer gait trajectories to the reference curve by reducing the error at 0.11 rad, suggesting potential benefits in the subsequent training efficacy (Zhang et al., 2022).

DT has been involved in two different tasks RC and LC. Thanks to their transparency even if is an old model it remains one of the most used and competitive. Thanks to the ability to treat categorical data they are particularly suitable for distinguishing between different locomotion types (Badesa et al., 2014). Usually, less amount of data is needed to train DT with respect to other techniques (Colledanchise and Ögren, 2018).

## 5 Discussion

The author deemed the results of the literature analysis reliable, as 60% of the papers originated from scientific journals with a peer-review process, while the remaining 40% were sourced from international conferences with peer-reviewed proceedings. Notably, all papers underwent a thorough revision process, further bolstering the validity of the findings.

Table 4 reports the paper presented in this review grouped by applications and AI algorithm implemented.

**TABLE 4:** Overview of the works analyzed in this review grouped by application of the AI-based algorithm.

	Robot control (RC)	Locomotion classification (LC)	Intention detection (ID)	Human joints trajectory prediction (HJTP)
Reinforcement Learning	Khan et al. (2019); Xu et al. (2020); Gao et al. (2020); Luo et al. (2021); Peng et al. (2021); Rose et al. (2022); Luo et al. (2023)			
Support Vector Machine	Lin et al. (2017)	Ceseracciu et al. (2010); Wu et al. (2016); Li et al. (2017); Ziegler et al. (2018); Wang et al. (2019); Ma et al. (2019); Ge et al. (2022)	Shen et al. (2013); Guo et al. (2020)	
Neural Network	Yang et al. (2019); Yingxu et al. (2019); Fereydooni et al. (2020); Shi et al. (2020); Wang et al. (2021b); Lin and Sie (2023)	Lim et al. (2010); Jung et al. (2015); Tang et al. (2021); Thongsook et al. (2019)		Luu et al. (2011); Liu et al. (2016); Błażkiewicz and Wit (2018); Xiong et al. (2019); Zhou et al. (2020); Wang et al. (2021b)
Decision Tree	He (2022); Zhang et al. (2022)	Guo and Jiang (2015); Ren et al. (2018); Thongsook et al. (2019); Imura et al. (2021)		

Analyzing the table by column we notice that in the area of RC, researchers have extensively explored RL and NN. RL has garnered significant attention, as evidenced by a substantial number of research papers. RL’s popularity can be attributed to its unique ability to enable exoskeletons to learn optimal control strategies through interactions with their environment. By receiving feedback in the form of rewards or penalties, RL algorithms iteratively improve their decision-making processes, leading to adaptable and robust control in complex and dynamic tasks. On the other hand, NNs have also been applied in the context of RC, albeit to a lesser extent compared to RL. These models are known for their capacity to approximate complex functions and effectively learn from high-dimensional data (Lin and Sie, 2023). When applied to RC tasks, NNs can capture intricate patterns and relationships in sensor data or articulation configurations, facilitating more nuanced and sophisticated control strategies. Both RL and NNs have showcased promising results in the field of RC. The choice of which algorithm to employ often depends on the specific requirements of the control task, with RL being favored in scenarios that necessitate adaptive learning and dynamic decision-making, while NNs are effective when dealing with high-dimensional sensor data and intricate control mappings. Overall, the combination of these two techniques showcases the ongoing advancements in using AI to enhance and refine exoskeleton control capabilities.

In the field of LC, researchers have employed three machine-learning techniques: SVM, NN, and DT. Among these techniques, SVM (Lin et al., 2017) has been particularly used for pneumatic locomotion. SVM’s use in this domain is limited. Still, it remains relevant due to its ability to effectively classify data in binary setting, and thanks to the “kernel trick” it is possible also to classify elements that originally aren’t linearly separable. Conversely, NN exhibits a more diverse range of applications in LC (Lin and Sie, 2023). NNs are well-suited for those tasks as they learn intricate relationships between input signals and locomotion types. By training on vast datasets, NNs can capture complex patterns and variations in human movement, leading to accurate and robust classification results. DTs, while less explored in the LC domain, are still represented by He (2022); Zhang et al. (2022). They offer interpretability, allowing researchers to gain insights into the decision-making process of the model. Additionally, DTs can effectively handle categorical data, making them suitable for classification tasks with discrete outcomes, such as distinguishing between different locomotion types. The three machine learning techniques used in LC each offer unique advantages: SVM for its effectiveness in handling non-linear classification tasks, NN for its ability to capture complex patterns, and DT for its interpretability and suitability for categorical data. The combination of these techniques showcases the diversity of approaches researchers employ to address the challenges of locomotion classification and further advance the field.

In the domain of ID, researchers have predominantly employed two primary machine learning techniques: RL and SVM. While both methods have been utilized, RL has been explored in a more limited number of papers (Guo et al., 2020; Imura et al., 2021). On the other hand, SVM is involved in more tasks (Wu et al., 2016; Ma et al., 2019). Intention detection plays a crucial role in human-robot interaction, as it enables exoskeletons to interpret human intentions and commands accurately. By employing RL in ID, researchers aim to create intelligent and adaptable systems that can learn from interactions with humans and optimize their decision-making processes accordingly. RL allows exoskeletons to understand human intentions through a continuous feedback loop, enhancing the effectiveness of human-robot communication and cooperation. Meanwhile, the higher representation of ID research highlights its efficacy in addressing classification tasks related to human intentions. SVM excels in binary classification, making it a suitable choice for discerning various human commands or intentions in real-time scenarios. Its ability to effectively classify data based on feature vectors from sensors or inputs further enhances its applicability in human-robot interaction. Overall, the combination of RL and SVM in intention detection research emphasizes the importance of developing accurate and reliable methods for enabling seamless and intuitive human-robot communication. As researchers continue to explore and refine these machine-learning techniques, the potential for advancing human-robot interaction and collaboration in various domains becomes increasingly promising.

In the field of HJTP, NN stands out as the primary machine learning technique employed, as indicated by the considerable number of cited papers up to Wang et al. (2021b). The prevalence of NNs in this domain is a testament to their remarkable ability to capture complex spatiotemporal patterns in human joint movements, making them particularly well-suited for trajectory prediction tasks. NNs excel in learning from large datasets and extracting meaningful representations from sequential data, which is critical for predicting the continuous and dynamic nature of human joint trajectories. By utilizing recurrent and convolutional architectures, NNs can effectively model the dependencies and interactions between joint positions over time, allowing them to forecast future joint movements accurately. The utilization of NNs in HJTP research signifies the growing recognition of their potential to enhance human-robot interactions, rehabilitation processes, and motion analysis. The ability to accurately predict human joint trajectories contributes to safer and more efficient human-robot collaboration, as the exoskeleton can anticipate and adapt to human movements in real-time. Moreover, the advancement of Neural Network architectures, such as LSTM networks and Transformer-based models, has further strengthened their predictive capabilities, enabling them to handle longer temporal dependencies and capture finer-grained patterns in joint movements. As HJTP continues to be a critical area of research, NN remains at the forefront of innovation, driving advancements in human-robot interaction, assistive robotics, and personalized rehabilitation. The ongoing developments in NNs promise to revolutionize how exoskeleton perceive and interact with human users, ultimately enhancing the overall performance and safety of human-robot collaborative tasks.

### 5.1 Conclusion and future direction

In synthesizing the key findings from our review, a prominent pattern emerges across the diverse landscape of exoskeleton-assisted lower-limb robot-aided rehabilitation. A prevalent observation within the literature is the recurring need for algorithm extensive validation on human subjects. While numerous promising solutions have been developed, a significant proportion of these works remain confined to simulation environments or solely validated on healthy individuals. This crucial step towards the real-world application of AI-driven rehabilitation solutions cannot be underestimated, particularly when considering their translation into clinical settings. The transition from a controlled experimental environment to the complex and dynamic conditions of a clinical context demands rigorous validation to ensure both the efficacy and safety of AI-based algorithmic solutions. The gap between simulation-based or healthy-subject validation and clinical implementation underscores a critical concern. It emphasizes that the bridge between research and practical clinical deployment necessitates robust human-centered validation studies, ideally involving individuals who are representative of the target patient population.

Moreover, a notable gap within the current literature pertains to the integration of human-centered technologies. While the research landscape showcases a wealth of technological advancements, a paucity exists in studies that holistically consider human opinions, preferences, and expert insights. The involvement of medical professionals, who hold a comprehensive understanding of patient needs and clinical requirements, is particularly vital. A critical challenge lies in ensuring that the developed AI technologies align seamlessly with medical practitioners’ perspectives, workflows, and patient care objectives. It becomes evident that not only validation but also the comprehensive consideration of human opinions and expert insights play pivotal roles. In the future design process of AI devices and methodologies, it is imperative to consistently prioritize the patient, who serves as the end-user of the technology. Considering and incorporating user feedback and assessing the impact that technology has on the individual is central to the development of person-centered technologies. This approach ensures that emerging technologies align closely with the needs and experiences of the end-users, fostering a more effective and user-friendly integration into healthcare practices.

Another important emerging point is that most of the existing datasets are currently private, while we encourage researchers and practitioners to make the data publicly available to sustain the research progress. In this respect, interested readers can refer to David et al. (2023) for a survey on the published datasets about human locomotion that, however, only in a few cases are ready to be used to train machine learning algorithms.

The survey of the works reported in the previous sections points out unsupervised learning has not been explored. Its use may likely be further studied in robot-assisted walking to pre-train supervised learning approaches (Erhan et al., 2010). Moreover, we notice that only three papers exploit deep-RL (Rose et al., 2022; Luo et al., 2023): we deem that investigating this learning paradigm is a promising direction for research also because deep-RL has shown its potential to outperform classical RL algorithms. For instance, this happens in sequential tasks since deep-RL can benefit LSTM architectures inside the RL paradigm (Li, 2017; Yu et al., 2020).

## 6 Conclusion

This paper presented a literature review on the use of artificial intelligence in the context of lower-limb robot-aided rehabilitation. This study identifies which algorithms are mainly used to address the different aspects such as robot control, walking pattern classification, motion interaction detection, and motion planning of the robotic system. It is worth noting that the devices currently used in clinical settings do not integrate AI algorithms into their functioning even if the scientific literature reviewed here has demonstrated how these methodologies may improve robot-assisted walking.

On the one side, this review also showed that some AI algorithms are suitable for solving specific problems, e.g., RL is only used for exoskeletal device control tasks, while other approaches are flexible to different application domains. On the other side, the take-home messages emphasize the need for the scientific community working with lower-limb exoskeletons to push forward the integration of AI methodologies in research programs and industrial applications. Considering the ethical integration of AI in lower-limb robot-aided rehabilitation demands a delicate balance between efficiency gains and preserving employment opportunities for therapists. Prioritizing patient safety through robust standards is imperative, and human supervision in automated decision-making ensures alignment with ethical standards. Transparency in AI algorithms, especially in safety exoskeleton development, is essential for building trust. Addressing algorithmic bias with human oversight is crucial to prevent disparities in treatment outcomes among diverse demographic groups. A collaborative, ethical approach is necessary to navigate these challenges and prioritize patient wellbeing in the evolving landscape of AI-driven healthcare (Durán and Jongsma, 2021; Kordzadeh and Ghasemaghaei, 2022; Morone et al., 2022).

## Figures and Tables

**Table:** 

**ADP**	Approximate Dynamic Programming
**AI**	Artificial Intelligence
**CPG**	Central Pattern Generator
**CGA**	Clinical Gait Analysis
**CoP**	Center of Pressure
**DRGL**	Deep Rehabilitation Gait Learning
**DT**	Decision Tree
**Emg**	Electromyography
**GAN**	Generative Adversarial Network
**H**	Healthy Prediction: HJTP
**HJTP**	Human Joints Trajectory Prediction
**ID**	Intention Detection
**KG-QL**	Knowledge-guided Q-learning
**KRLS**	Kernel Recursive Least Squares
**LC**	Locomotion Classification
**LLRER**	Lower-Limb Robotic Exoskeleton Robot
**LSTM**	Long Short-Term Memory
**MLP**	Multilayer Perceptron
**MR**	Magnetorheological
**MRAC**	Model Reference Adaptive Controller
**NARX**	Nonlinear Autoregressive Exogenous Model
**NRMSE**	Normalized Root-Mean-Square Error
**NN**	Neural Network
**P**	Pathological
**PSO**	Particle Swarm Optimization
**RBF**	Radial Basis Function
**RC**	Robot Control
**RL**	Reinforcement Learning
**RNN**	Recurrent Neural Network
**sEMG**	Surface Electromyography
**Sim**	Simulated
**SVM**	Support Vector Machine
**VIC**	Variable Impedance Controller
**WNN**	Wavelet Neural Network
